# Biomechanical studies of different numbers and positions of cage implantation on minimally invasive transforaminal interbody fusion: A finite element analysis

**DOI:** 10.3389/fsurg.2022.1011808

**Published:** 2022-11-07

**Authors:** Zhenchuan Han, Chao Ma, Bo Li, Bowen Ren, Jianheng Liu, Yifei Huang, Lin Qiao, Keya Mao

**Affiliations:** ^1^ Department of Orthopedics, Chinese PLA General Hospital, Beijing, China; ^2^ Department of Orthopedics, PLA Rocket Force Characteristic Medical Center, Beijing, China; ^3^ Key Laboratory of Modern Measurement and Control Technology, Ministry of Education, Beijing Information Science and Technology University, Beijing, China; ^4^ Department of Orthopedics, Weihai Municipal Third Hospital, Weihai, China; ^5^ Department of Orthopedics, The Fourth Affiliated Hospital of Xinjiang Medical University, Urumqi, China

**Keywords:** CAGE, finite element analysis, MIS-TLIF, biomechanical, lumbar fusion

## Abstract

**Background:**

The position and number of cages in minimally invasive transforaminal interbody fusion (MIS-TLIF) are mainly determined by surgeons based on their individual experience. Therefore, it is important to investigate the optimal number and position of cages in MIS-TLIF.

**Methods:**

The lumbar model was created based on a 24-year-old volunteer's computed tomography data and then tested using three different cage implantation methods: single transverse cage implantation (model A), single oblique 45° cage implantation (model B), and double vertical cage implantation (model C). A preload of 500 N and a moment of 10 Nm were applied to the models to simulate lumbar motion, and the models' range of motion (ROM), ROM ratio, peak stress of the internal fixation system, and cage were assessed.

**Results:**

The ROM ratios of models A, B, and C were significantly reduced by >71% compared with the intact model under all motions. Although there were subtle differences in the ROM ratio for models A, B, and C, the trends were similar. The peak stress of the internal fixation system appeared in model B of 136.05 MPa (right lateral bending), which was 2.07 times that of model A and 1.62 times that of model C under the same condition. Model C had the lowest cage stress, which was superior to that of the single-cage model.

**Conclusion:**

In MIS-TLIF, single long-cage transversal implantation is a promising standard implantation method, and double short-cage implantation is recommended for patients with severe osteoporosis.

## Introduction

**Figure 1:**
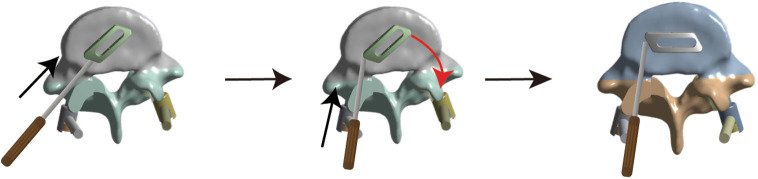
Diagram of cage traverse implantation.

Lumbar degenerative disease (LDD) is a major cause of intractable low back and leg pain in middle-aged and older people (1). Interbody fusion is the standard surgical procedure for treating persistent neurological symptoms caused by LDD when conservative treatment fails (2). Minimally invasive transforaminal interbody fusion (MIS-TLIF), first reported by Professor Foley in 2003 (3), has been widely used as a minimally invasive fusion method to treat LDD (4–6). Compared with traditional open surgery such as posterior lumbar interbody fusion (PLIF) or transforaminal lumbar interbody fusion (TLIF), MIS-TLIF can significantly reduce surgical trauma, bleeding, postoperative pain, and infection and greatly preserve the physiological function of muscles (7). Interbody fusion is one of the most challenging technical aspects of MIS-TLIF. Currently, interbody fusion is mainly processed by cage implantation, which plays an important role in vertebral body fusion as a permanent implantation (8). Currently, controversies remain regarding the number and position of MIS-TLIF surgical fusion cages in clinical practice. Some reports advocate the application of double-cage implantation in the intervertebral space in MIS-TLIF (9, 10) whereas others demonstrate that single oblique cage implantation can provide sufficient mechanical support (11–13). Recently, a few scholars have innovatively proposed placing a single cage parallel to the posterior longitudinal ligament in the intervertebral space and have achieved good clinical results (14, 15). The implantation method was as follows: first, the cage was inserted at 45°, and then the end of the cage was knocked to make it rotate horizontally (Figure 1). Therefore, it is important to investigate the optimal number and position of fusion cages implanted in MIS-TLIF.

Analysis of lumbar spinal biomechanical properties is an ongoing challenge because of the complex shapes and heterogeneous biological structures of the human lumbar spine. The finite element (FE) model is ideal for evaluating spinal biomechanics because it is not affected by complicated clinical factors and provides detailed data that cannot be obtained by experimental methods (16, 17). Herein, the FE model of the lumbar spine was used to evaluate the effect of the different numbers and positions of cages in the intervertebral space on lumbar spinal biomechanics, hopefully providing clinicians with surgical references and promoting the standardization of the MIS-TLIF surgical cage.

## Materials and methods

### Fe models of the lumbar spine

The lumbar model was created based on the computed tomography (CT) data of a healthy volunteer. The volunteer provided written informed consent, although his data were anonymized and local hospital trust ethical policies were adhered to. Lumbar CT images of a healthy 24-year-old male volunteer (70 kg, 176 cm, no history of lumbar spine disease) were collected with an image interval of 0.625 mm (Philips Brilliance 64 Slice CT, Philips Medical Systems, Inc., OH, USA), and data were saved in DICOM format. These images were then imported into Mimics Research 19.0 (Materialise, Inc.) software to preprocess the CT images and obtain the L4–L5 preliminary three-dimensional geometric model. Subsequently, a file (in.stl format) generated by Mimics was imported into the Geomagic Wrap 2017 (3D Systems, Inc.) software to optimize and smooth the model. This file (.stl format) generated by Geomagic was further imported into Solid Works 2017 (Dassault Systems, Inc.) software to combine and assemble the bones, annulus, nucleus pulposus, screws, and cages, followed by generating a reconstructed model.X_T file. Finally, the.X_T file was imported into ANSYS V20.0 software (ANSYS, Inc.) for FE analysis (FEA).

**Figure 2:**
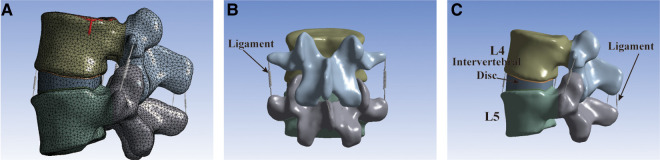
The intact L4–L5 segment FE model. (**A**) The model utilized tetrahedral elements for FE meshing. (**B**) Front view of the FE model. (**C**) Lateral view of the FE model.

The model utilized tetrahedral elements for FE meshing, except for the ligaments (Figure 2A). There were ligaments around the lumbar vertebral body that could limit the range of motion (ROM) of the vertebral body of the spine. However, because the ligament model was too slender and irregular in shape, a spring element was used in the model to simulate the ligament of the intervertebral body (Figures 2B,C). The ligaments of the lumbar spine were as follows: anterior longitudinal ligament, posterior longitudinal ligament, ligamentum flavum, interspinous ligament, supraspinous ligament, intertransverse ligament, and joint capsule ligament. The vertebral body was divided into the outer cortical bone and the inner cancellous bone. The thickness of the cortical bone was 1.0 mm and that of the bone endplate was 0.5 mm (18), and the endplates were set on the superior and inferior surfaces of each vertebral body. The intervertebral discs were divided into nucleus pulposus and annulus fibrosus. The interfacing of the nucleus pulposus and annulus fibrosus and the interfacing of the disc and vertebral body were set as binding. The interfaces of the vertebrae and cages were also assigned to tie constraints (18). The material properties were determined as previously reported (19, 20). Finally, Young's modulus, Poisson's ratio, cross-sectional area, and other data (Table 1) of the materials were inputted to complete the establishment of the intact L4–L5 segment FE model (model INT).

**Table 1:** Material properties used in the present finite-element model of the lumbar spine.

Material properties	Young's modulus, MPa	Poisson's ratio, m	Cross-sectional area, mm^2^
Endplate	12,000	0.3	–
Posterior bone	3,500	0.25	–
Articular cartilage	25	0.25	–
Annulus fibrosus	6	0.40	–
Nucleus pulposus	1	0.50	–
Cortical bone	12,000	0.3	–
Cancellous bone	100	0.2	–
ALL	7.8	–	22.4
PLL	1	–	7.0
LF	1.7	–	14.1
ITL	1	–	0.6
CL	7.5	–	10.5
ISL	1	–	14.1
SSL	8	–	10.5
Pedicle screws and rods (titanium alloy material)	110,000	0.3	
Cage (PEEK material)	3,500	0.3	

ALL, anterior longitudinal ligament; PLL, posterior longitudinal ligament; LF, ligamentum flavum; ISL, interspinous ligament; SSL, supraspinous ligament; ITL, intertransverse ligament; CL, joint capsule ligament.

### Fe models of the MIS-TLIF

The L4–L5 functional spinal unit was selected to evaluate the MIS-TLIF technique, as it is the most frequent site of LDD requiring surgical treatment (21). The unilateral or bilateral nerve decompression approach was selected based on the number of cages implanted. The steps of the MIS-TLIF procedure are as follows: First, unilateral or bilateral L5 upper articular process, left or bilateral L4 lower articular process, ligamentum flavum, and posterolateral annulus fibrosus were removed. The nucleus pulposus tissues and cartilage endplates in the intervertebral disc were then removed. The experimental simulation of the intervertebral fusion cage was based on a Z-cage (WeGo Company, Shandong, China) with dimensions of 32 × 10 × 12 mm (single-cage implantation) and 22 × 10 × 12 mm (double-cage implantation). The cage material used was polyether ether ketone (PEEK, *E* = 3.6 GPa). The internal fixation system simulated in the experiment was modeled using the Premier System (WeGo Company, Shandong, China). The screw was 45 mm in length, with a diameter of 6.0 mm, and the connecting rod was 40 mm in length, with a diameter of 5.5 mm. All the materials were made of titanium alloy (*E* = 110 Gpa).

**Figure 3:**
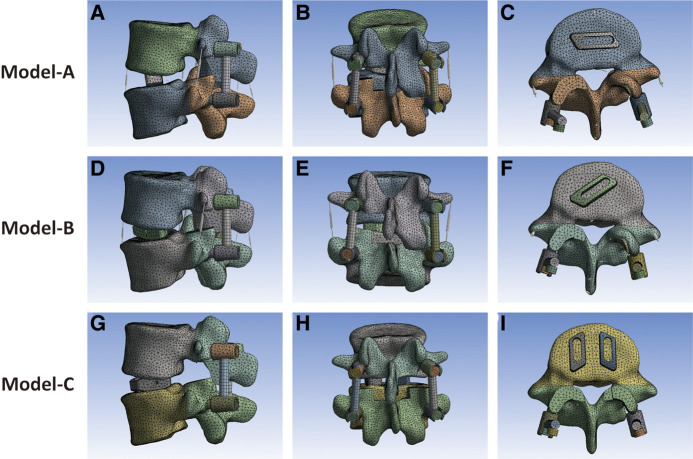
(**A–C**) Single 32 × 10 × 12 mm fusion cage horizontal implantation model. (**D–F**) Single 32 × 10 × 12 mm fusion cage oblique 45° implantation model. (**G–I**) Double 22 × 10 × 12 mm fusion cages vertical implantation model.

Based on the number and position of cage implantation, the MIS-TLIF surgical models were divided into three groups: model A, single-cage (32 × 10 × 12 mm) transverse implantation model (Figures 3A–C); model B, single-cage (32 × 10 × 12 mm) oblique 45° implantation model (Figures 3D–F); and model C, double-cage (22 × 10 × 12 mm) vertical implantation model (Figures 3G–I).

### Boundary and loading conditions

The lower endplate of L5 was fixed with zero degrees of freedom to ensure that there was no displacement or rotation of L5 under external forces. A 500 N preload was vertically applied to the upper endplate of L4 to simulate the upper body weight (16). Additionally, a 10 N/m force was applied to simulate the physiological activities of the lumbar spine, such as flexion (FL), extension (EX), left lateral bending (LLB), right lateral bending (RLB), left rotation (LR), and right rotation (RR) (16). Furthermore, the ROM of the lumbar spine, peak stress, and average stress of the internal fixation system and cages under various working conditions were recorded and analyzed. To compare the ROM between models, the ROM ratio was calculated using model INT as the reference: [(model INT − model A/B/C) ÷ model INT] × 100% (22).

## Results

### Model validation

**Figure 4:**
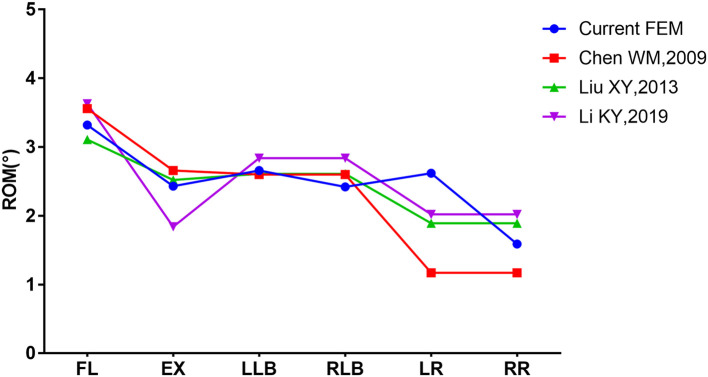
Range of motion of the model compared with literature reports.

The ROM of model INT was compared with the research results of Chen (23), Liu (24), and Li (25) by applying the same loads to our model. The results confirmed the effectiveness of our model, as shown in Figure 4.

### ROM

The ROM and ROM ratios of model INT, model A, model B, and model C under different conditions are listed in Table 2. The ROM ratio of the fused L4–L5 segments was significantly reduced by more than 71% compared with that of model INT under all motions. In the single-cage model, the model with a transversely implanted cage showed superior stability, and the ROM ratio of model A was higher than that of model B in almost all motions, except for LLB motion (80.83% in model A vs. 81.20% in model B). Compared with the single-cage models, the double-cage model displayed superior stability: the ROM ratio of model C was higher than that in model A and model B in almost all motions, except for the EX motion (93.42% in model C vs. 97.53% in model A vs. 96.71% in model B).

**Table 2:** Lumbar spine range of motion under each working condition of the four models.

Direction of motion	Model INT (°)	Model A (°)/[ROM ratio]	Model B (°)/[ROM ratio]	Model C (°)/[ROM ratio]
FL	3.32	0.79	(76.20%)	0.94	(71.69%)	0.70	(78.92%)
EX	2.43	0.06	(97.53%)	0.08	(96.71%)	0.16	(93.42%)
LLB	2.66	0.51	(80.83%)	0.50	(81.20%)	0.39	(85.34%)
RLB	2.42	0.48	(80.17%)	0.66	(72.73%)	0.48	(80.17%)
LR	2.62	0.38	(85.50%)	0.46	(82.44%)	0.28	(89.31%)
RR	1.59	0.43	(72.96%)	0.46	(71.07%)	0.28	(82.39%)

FL, lumbar flexion; EX, extension; LLB, left lateral bending; RLB, right lateral bending; LR, left rotation; RR, right rotation. ROM ratio = (Model INT − Model A/B/C/D) ÷ Model INT × 100%.

### Peak stress and average stress of the internal fixation system

**Figure 5:**
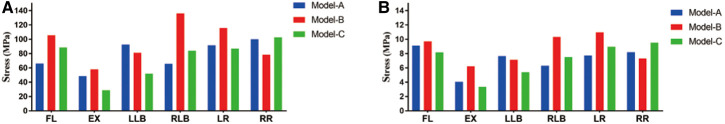
(**A**) Peak stress of the internal fixation system. (**B**) Average stress of the internal fixation system.

The peak stresses of the internal fixation system under different motions are shown in Figure 5A. The highest peak stress of the internal fixation system was 136.05 MPa featured in model B in RLB motion, which is 2.07 times that of model A (65.68 MPa) and 1.62 times that of model C (84.07 MPa) under the same conditions. For the single-cage model, the peak stress of the internal fixation system of model A was significantly lower than that of model B under the four motions of FL, EX, RLB, and LR, which were 65.93 vs. 105.60 MPa, 48.56 vs. 58.10 MPa, 65.68 vs. 136.05 MPa, and 91.55 vs. 115.98 MPa, respectively. Compared with the double-cage model, the peak stress of model C was lower than that of model B in five motions, but the peak stresses of models C and A were comparable. The average stress on the internal fixation system is shown in Figure 5B. The average stress of model B was also significantly higher than those of models A and C under four motions (FL, EX, RLB, and LR). The average stresses of models A and C exhibited comparable trends.

### Stress cloud diagram, peak stress, and average stress of cage

**Figure 6:**
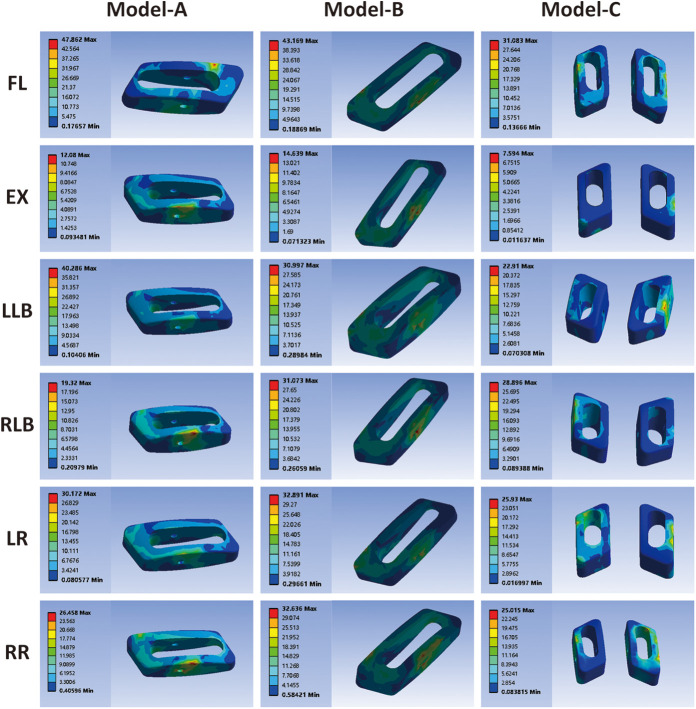
Stress cloud diagram of the cage.

**Figure 7:**
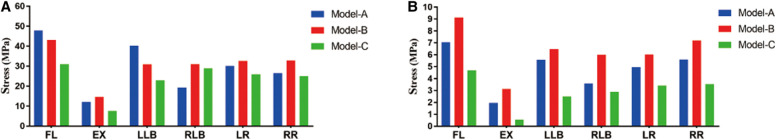
(**A**) Peak stress of the cage. (**B**) Average stress of the cage.

As shown in Figure 6, the stress cloud diagram of the cage exhibits different peak stress positions under different conditions. Peak stress develops at the area of contact between the cage and vertebral endplate. The peak stress in the cage predicts the stress on the endplate owing to the interaction between these forces. The peak stresses of the cages of the three models are shown in Figure 7A. For the single-cage model, model A cage exhibited higher peak stresses than model B cage in FL (47.86 vs. 43.17 MPa) and LLB (40.29 vs. 31.00 MPa) motions, but displayed lower peak stresses in EX, RLB, LR, and RR motions. Compared with single-cage models, the double-cage model showed superiority for the peak stress: the peak stress of model C was lower than those of models A and B in almost all motions, except for the RLB motion. The average stress values of the different models are shown in Figure 7B. For the single-cage model, the average stress of model A was lower than that of model B under all motions. Meanwhile, the double-cage model exhibited lower average stress than the single-cage model under all motions, shown as 4.69 MPa in FL, 0.55 MPa in EX, 2.49 MPa in LLB, 2.88 MPa in RLB, 3.41 MPa in LR, and 3.53 MPa in RR.

## Discussion

MIS-TLIF has shown remarkable advantages and has become a primary minimally invasive therapeutic method in treating LDD since its application (6, 26). Currently, spinal fusion cages are widely used in MIS-TLIF surgery to maintain intervertebral disc height, promote bony fusion, and restore lumbar lordosis (27). However, cage-related complications have been reported, such as cage migration and cage subsidence (28). Studies have proved that the stress shielding, shape, and position of the cage in the intervertebral space are significant factors affecting cage displacement (8, 29). The number and position of implanted fusion cages in clinical practice are yet to be determined by surgeons based on their individual experience. Therefore, we conducted this FE study to provide biomechanical evidence for surgeons to determine the number and position of the implanted cages in MIS-TLIF.

For the choice of cages in the model, we used a single cage with a diameter of 32 mm and double cage with a diameter of 22 mm. A study reported that a cage with a longer diameter has a larger contact area with the endplate, which can promote bony fusion and reduce the risk of cage subsidence (30). Therefore, for single-cage implantation, we tended to choose cages with a longer diameter. Owing to the limitation of the intervertebral space, it is impractical to implant two longer-diameter cages; thus, surgeons often choose to implant two shorter-diameter cages, of which the 22 mm-diameter cage is the most commonly used.

The overall stability of the model was evaluated by measuring the ROM of the lumbar spine in each model (31). Compared with model INT, the MIS-TLIF model significantly reduced the range of activities by at least 71%. Biomechanical stability is consistent with clinical experience and previous research conclusions (18). A comparison of ROM ratios between models showed that the transverse cage model was more stable than the oblique 45° cage model. The dual-cage implantation model displayed better stability than the single-cage implantation model. Although the internal fixation system contributed the most to the stability of models (32, 33), the difference in the number and position of the implanted cages also affected the stability of the models, as observed in FEA. Theoretically, the double cage has a larger contact area with the endplate than the single cage, which enhances the frictional resistance between the cage and endplate, leading to increased stability.

As shown in the FEA of the internal fixation system, the peak stress of the internal fixation system occurred in the single-cage oblique 45° model, which was 2.07 times (in RLB) that of the single-cage transverse model and 1.62 times (in the RLB) that of the double-cage model under the same conditions. Moreover, the single-cage transverse model displayed a smaller peak stress in the internal fixation system than the single-cage oblique 45° model under multiple motions. In addition, the single-cage transverse model and double-cage model exhibited similar mechanical properties in terms of the peak stress and average stress of the internal fixation system. Therefore, internal fixation breakage is more likely to occur in the single-cage oblique 45° model than in the other models if the fusion segment is not effectively fused with the interface bone.

The high stress of the cage may cause cage migration or subsidence, resulting in the loss of intervertebral disc height and failure of the operation (34). By comparing the peak stress and average stress of the cage, it can be seen that double-cage models had the lowest cage stress, which was superior to that of single-cage models. Therefore, double-cage method is particularly suitable for patients with severe osteoporosis and can reduce the risk of cage sinking. When a double-cage method is applied, it is often difficult to place the cage symmetrically, and its head ends are prone to collide with each other, which increases the difficulty of the operation in the implantation process. Moreover, double-cage insertion inevitably causes excessive damage to the posterior stability of the spine, and these factors cannot be ignored. However, it is also important to note that compared with single-cage implants, double-cage implants can prolong operative time and increase bleeding and medical costs (35). For single-cage models, the average stress of the single-cage transverse model was lower than that of the single-cage oblique 45° model under all motions. It can be inferred that the subsidence risk of single-cage transverse implantation is lower than that of single-cage oblique 45° implantation. Theoretically, the risk of cage migration into the spinal canal is reduced in the single-cage transverse model because the cage is placed in the intervertebral space parallel to the posterior longitudinal ligament, which makes it difficult to withdraw and displace the cage. Therefore, based on the above reasons, we recommend that patients without osteoporosis obtain greater benefits with single-cage transverse implantations.

This study has some limitations this study. First, the FE model was constructed using CT images from healthy subjects without any spinal disease. Therefore, changes in the geometry of the spine and implantations were not considered. Second, biomechanical changes in adjacent segments were not evaluated in this study because intervertebral fusion can lead to adjacent segment degeneration. Finally, the paraspinal muscles were not considered in the entire investigation, which could slightly affect the stability of the lumbar spine.

## Conclusion

According to the FEA results, the number and position of the cage in MIS-TLIF significantly influence the biomechanics of the lumbar spine. Double-cage implantation exhibits excellent biomechanical properties in terms of spinal stability and stress distribution in the internal fixation system and the cage. However, the advantages of the single-cage transverse model are excellent, and its safety and effectiveness have been verified clinically. Therefore, patients with severe osteoporosis should choose the double-cage implantation method, whereas the single-cage transverse implantation method is recommended for patients without osteoporosis, which could be a promising standard for cage implantation in MIS-TLIF.

## Data Availability

The datasets presented in this study can be found in online repositories. The names of the repository/repositories and accession number(s) can be found in the article/Supplementary Material.
